# Enumeration, functional responses and cytotoxic capacity of MAIT cells in newly diagnosed and relapsed multiple myeloma

**DOI:** 10.1038/s41598-018-22130-1

**Published:** 2018-03-07

**Authors:** Nicholas A. Gherardin, Liyen Loh, Lorenztino Admojo, Alexander J. Davenport, Kelden Richardson, Amy Rogers, Phillip K. Darcy, Misty R. Jenkins, H. Miles Prince, Simon J. Harrison, Hang Quach, David P. Fairlie, Katherine Kedzierska, James McCluskey, Adam P. Uldrich, Paul J. Neeson, David S. Ritchie, Dale I. Godfrey

**Affiliations:** 1 Department of Microbiology and Immunology, Peter Doherty Institute for Infection and Immunity, University of Melbourne, Parkville, Victoria 3010 Australia; 2 Cancer Immunology Program, Peter MacCallum Cancer Centre, Melbourne, Victoria 3000 Australia; 3 ARC Centre of Excellence in Advanced Molecular Imaging, University of Melbourne, Parkville, Victoria 3010 Australia; 4 The Walter and Eliza Hall Institute of Medical Research, Parkville, Victoria 3050 Australia; 5 Clinical Haematology and Bone Marrow Transplant Service, Royal Melbourne Hospital, Parkville, Victoria 3002 Australia; 6 Division of Chemistry & Structural Biology, Institute for Molecular Bioscience, The University of Queensland, Brisbane, Queensland 4072 Australia; 7 ARC Centre of Excellence in Advanced Molecular Imaging, University of Queensland, Queensland, 4072 Australia; 8 Department of Medicine, University of Melbourne, Parkville, Victoria 3010 Australia; 9 Sir Peter MacCallum Department of Oncology, The University of Melbourne, Parkville, 3010 Australia

## Abstract

Mucosal-associated invariant T (MAIT) cells are T cells that recognise vitamin-B derivative Ag presented by the MHC-related-protein 1 (MR1) antigen-presenting molecule. While MAIT cells are highly abundant in humans, their role in tumour immunity remains unknown. Here we have analysed the frequency and function of MAIT cells in multiple myeloma (MM) patients. We show that MAIT cell frequency in blood is reduced compared to healthy adult donors, but comparable to elderly healthy control donors. Furthermore, there was no evidence that MAIT cells accumulated at the disease site (bone marrow) of these patients. Newly diagnosed MM patient MAIT cells had reduced IFNγ production and CD27 expression, suggesting an exhausted phenotype, although IFNγ-producing capacity is restored in relapsed/refractory patient samples. Moreover, immunomodulatory drugs Lenalidomide and Pomalidomide, indirectly inhibited MAIT cell activation. We further show that cell lines can be pulsed with vitamin-B derivative Ags and that these can be presented via MR1 to MAIT cells *in vitro*, to induce cytotoxic activity comparable to that of natural killer (NK) cells. Thus, MAIT cells are reduced in MM patients, which may contribute to disease in these individuals, and moreover, MAIT cells may represent new immunotherapeutic targets for treatment of MM and other malignancies.

## Introduction

Multiple myeloma is a haematological malignancy characterised by a clonal outgrowth of malignant plasma cells in the bone marrow^1^. Advances in stem cell transplants and therapeutics over the last 15 years have seen major improvements in the long-term survival and quality of life of patients diagnosed with MM^2^, yet despite these advances, MM remains an incurable disease, with a median survival around 7 years^1^.

The current treatment of MM is largely based on the doublet or triplet combinations of corticosteroids, proteasome inhibitors and/or immunomodulatory drugs (IMiDs) as induction therapy prior to autologous stem cell transplantation in younger patients. Lenalidomide (Len), is the most extensively used IMiD for the treatment of MM^3^. It has both direct anti-tumor and immune-mediated mechanisms of action through binding cereblon (CRBN), a component of an E3-ubiquitin ligase^4^. Len commonly forms the backbone of newly developed combination immune therapies in MM, which now include the addition of monoclonal antibodies as initial therapy or as salvage therapy in the relapsed disease setting^5–8^. Given the increasing importance of immunological cellular effectors in the mechanisms of action of these emergent therapies, a greater understanding of immune function in patients with MM will enhance the application and development of future immune-based therapies.

Whereas conventional peptide-MHC-reactive T cells are the main focus of many studies into cancer immunotherapy, there is growing interest in the potential to harness unconventional T cells that are not peptide-MHC reactive. These studies include, for example, modulation of CD1d-lipid-antigen (Ag)-restricted type I NKT cells using the lipid-Ag α-galactosylceramide^9^, and the phospho-Ag-reactive Vγ9Vδ2 γδ T cells using bisphosphonates such as zoledronic acid^10^. Compared to their peptide-MHC-restricted conventional T cell counterparts, the high precursor frequency, restriction to monomorphic antigen-presenting molecules, and powerful immunomodulatory properties give unconventional T cells great potential as a therapeutic target^11,12^. While these approaches have shown promise in pre-clinical models, and responses have been observed in early clinical trials including in MM^13^, our understanding of how these T cells behave in the context of this disease is very limited.

MAIT cells are highly abundant in humans, making up as much as 10% of the circulating T cell pool in healthy individuals^14^. MAIT cells are defined by expression of a semi-invariant TCR that pairs a conserved TCR-α chain (TRAV1-2-TRAJ33/12/20) with a constrained repertoire of TCR-β chains^15–17^ and their ability to recognise vitamin-B-related Ags presented by MHC-related protein 1 (MR1)^18^, a monomorphic Ag-presenting molecule with high sequence homology to MHC class I^19^. While MR1 can bind derivatives of both folate (vitamin B9) and riboflavin (vitamin B2), MAIT cells are universally and potently activated by the Ag 5-(2-oxopropylideneamino)-6-D-ribitylaminouracil (5-OP-RU), a chemical metabolite formed as a byproduct of microbial biosynthesis of riboflavin through condensation of a biosynthetic intermediate with the glycolysis metabolite pyruvaldehyde^20^.

Upon recognition of MR1-Ag complexes via their TCR, MAIT cells rapidly produce proinflammatory cytokines such as IFNγ and TNF^21^, and are capable of cytolytic activity^22^, and are thus emerging as important players in anti-microbial immunity and barrier defense^23^. The extent to which MAIT cells contribute to immunity in non-infectious diseases is unclear, although recent studies suggest that MAIT cell numbers and function are modulated in the setting of several diseases, including autoimmunity, metabolic disorders, and solid cancers^24,25^. While microbial antigens might not be involved in these settings, MAIT cells are also capable of responding via non-TCR mediated mechanisms such as in the case of viral infection, where they respond to IL-12 and IL-18^26^.

In the setting of haematological malignancy, MAIT cells remain largely unexplored. However, their high abundance, proinflammatory activity and cytolytic potential, as well as the ubiquitous expression and monomorphic nature of MR1, highlights the importance of understanding the role that these cells play, as MAIT cells may represent good candidates for immunotherapeutic manipulation in these diseases.

In this study we characterise MAIT cells in patients with MM. We show that in general, MM patients have very low levels of MAIT cells in their peripheral blood, which may largely reflect the age of these patients. There was also no evidence they were accumulating at the disease site (bone marrow) in these patients, in contrast to other inflammatory diseases where MAIT cells migrate from blood to disease location^27–32^. Moreover, the residual MAIT cells in these patients have reduced functional capacity, and this seems to be restored in relapsing-remitting MM patients following therapy. Furthermore, MM cell lines pulsed with the MAIT cell Ag 5-OP-RU can be efficiently and specifically lysed by *in vitro*-expanded MAIT cells in a TCR and MR1-dependent manner. These results suggest that MAIT cell deficiency may contribute to the impaired immunity in MM patients, and moreover, that manipulation of the MR1-Ag-MAIT cell axis may provide a novel avenue for immunotherapy in MM and other haematological malignancies.

## Results

### Low MAIT cell frequency in MM patients

**Figure 1:**
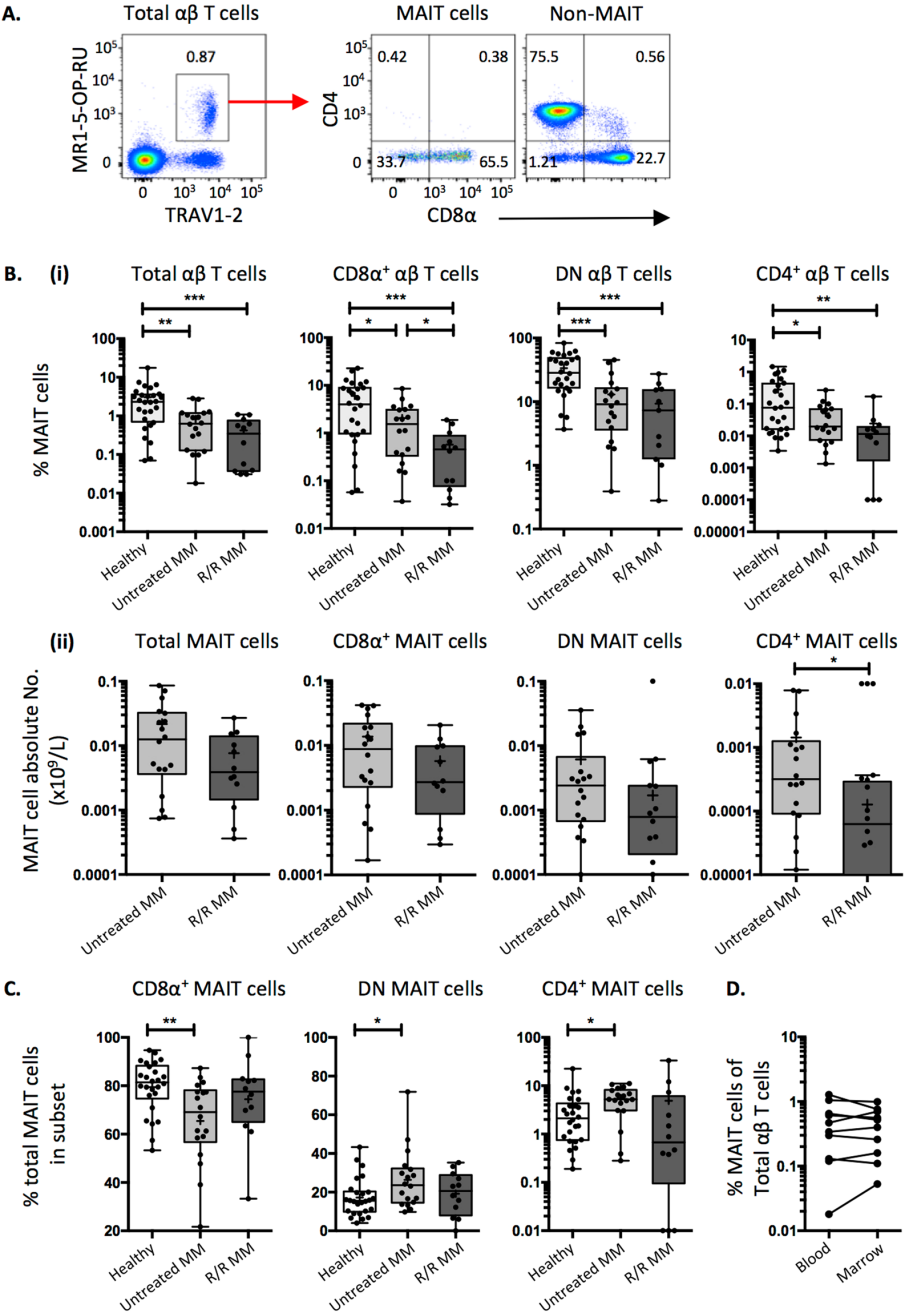
Analysis of MAIT cell frequency across patient cohorts. (**A**) Flow cytometric pseudocolour plots showing example MR1-5-OP-RU tetramer staining on PBMCs (left panel) and CD4/CD8α co-receptor distribution on MAIT cells versus non-MAIT T cells (middle and right panels) (**B**). Box and whisker plots showing: (i) the proportion and (ii) absolute numbers of MAIT cell subsets across patient cohorts. (**C**) Box and whisker plots showing the proportion of MAIT cells in T cell subsets defined by CD4/CD8α co-receptor expression. (**D**) Dot plots showing matched-pair analysis of the MAIT cell frequency in untreated myeloma patient peripheral blood and bone marrow (white boxes = healthy donors; light grey boxes = untreated multiple myeloma (MM) patients; dark grey boxes = refractory relapsed (R/R) MM patients).

Using MR1–5-OP-RU tetramers^17^ in combination with an antibody directed against TRAV1-2^+^ TCRs, MAIT cells were identified via flow cytometry (Fig. 1A) which allowed their enumeration in peripheral blood (PB). Two cohorts of MM patients: untreated, newly diagnosed patients and; refractory or relapsed (R/R) MM patients were compared to a cohort of healthy donors (Figure S1A). As expected, MAIT cells were abundant in healthy donors (mean 3.04%, range 0.07–17.6%), but untreated MM patients had a significantly lower proportion of MAIT cells in their peripheral blood (mean 0.83%, range 0.02–2.8%), and this was even more pronounced in R/R patients (mean 0.42% range 0.03–1.1) (Fig. 1Bi). This reduction in MAIT cell proportion was apparent regardless of whether it was expressed as a percentage of total αβ T cells, CD8^+^ T cells, DN T cells or CD4^+^ T cells (Fig. 1Bi). While the differences were generally not significant between the untreated and R/R patients for DN T cells, there was a significant reduction in MAIT cells as a proportion of CD8^+^ or CD4^+^ T cells between these two cohorts (Fig. 1Bi). The differences for CD4^+^ T cells were also reflected by the absolute number of circulating MAIT cells (Fig. 1Bii). The frequency of CD4^+^, DN and CD8^+^ subsets of MAIT cells, defined through antibody gating, showed a moderate but significant reduction in the proportion of CD8^+^ MAIT cells and a reciprocal increase in DN and CD4^+^ MAIT cells between healthy and untreated MM patients, that was not apparent in the R/R cohort (Fig. 1C). Taken together, these data suggest that MM patients have very low levels of circulating MAIT cells, with some MAIT cell subsets differentially affected in untreated and R/R disease.

Recent studies have shown that MAIT cell frequencies decline as a function of age^33,34^. While we did not have access to the ages of all of the healthy donors included in this study, the patients in the untreated and R/R MM cohorts were relatively older than might be expected from the healthy blood donors, with mean ages of 56 and 63 and ranges of 43–66 and 58–77 respectively (Figure S1A). To determine the influence of old age on the observed decline in MAIT cells in the MM patients, the above cohort of untreated MM patients was compared with an age-matched cohort of healthy donors (Figure S1B). While the mean and median percentages of MAIT cells, as a proportion of total T cells, CD8^+^, DN and CD4^+^ T cells, were lower in this group of MM patients compared to the age matched controls, this only reached statistical significance for CD4^+^ T cells (Figure S1C). These data suggest that the typically older age of MM patients may be a contributing factor at least for blood MAIT cells, but the reduced number of MAIT cells in these patients does not appear to be solely age-related.

In MM, the disease burden is largely confined to the bone marrow (BM). To determine whether MAIT cells infiltrate the tumour bed, we analysed BM aspirates from the untreated MM patients and compared them to matched PB. The proportion and co-receptor usage of MAIT cells in BM was largely reflective of peripheral blood suggesting that MAIT cells are not retained at the site of disease (Fig. 1D and data not shown).

### Reduced CD27 expression by MAIT cells in untreated MM patients

**Figure 2:**
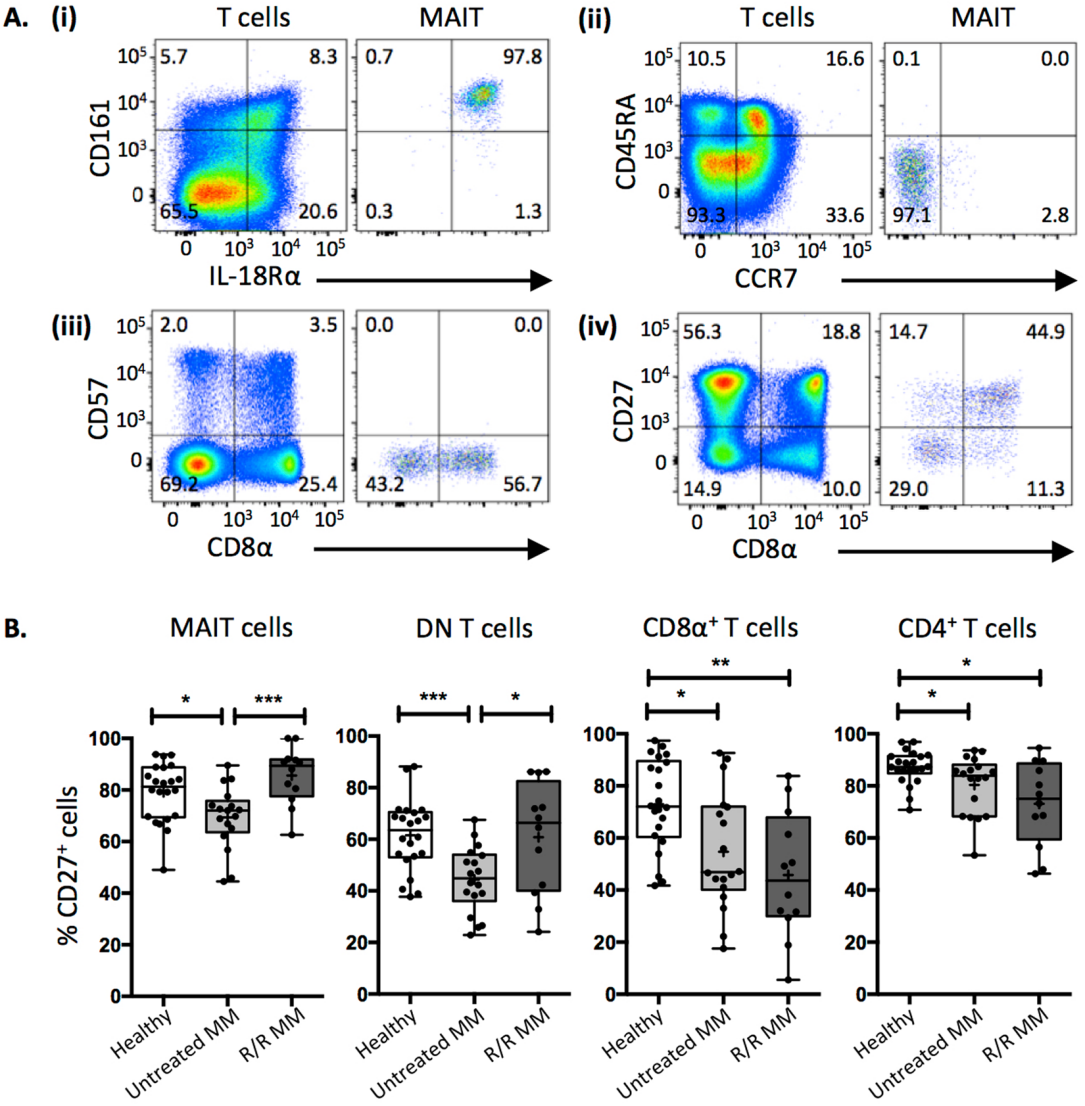
Analysis of MAIT cell phenotype. (**A**) Representative flow cytometric pseudo-colour plots of non-MAIT T cells (left panels) and MAIT cells (right panels) showing: (i) CD161 and IL-18Rα expression, (ii) CCR7 and CD45RA expression, (iii) CD57 expression, and (iv) CD27 expression, on a MM patient PBMCs. (**B**) Box and whisker plots showing the proportion of CD27^+^: MAIT cells, DN T cells, CD8α^+^ T cells, and CD4^+^ T cells across patient cohorts. (white boxes = healthy donors; light grey boxes = untreated multiple myeloma (MM) patients; dark grey boxes = refractory relapsed (R/R) MM patients). CD4^+^ and CD8^+^ T cell CD27 expression in figure B was previously published^37^, and is reproduced here for comparison.

A more extensive analysis of the cell surface phenotype of MAIT cells was also performed with samples from these patients, examining a series of markers known to be expressed by MAIT cells in healthy tissue. Consistently high levels of both CD161 and CD218 (IL-18Rα) were expressed by MAIT cells in all three cohorts (Fig. 2Ai and data not shown). Moreover, MAIT cells were largely negative for CD45RA and CCR7, consistent with an effector memory phenotype (Fig. 2Aii and data not shown).

CD57 is a marker of terminal differentiation on CD8^+^ T cells, and clonal/oligoclonal expansions of CD8^+^ CD57^+^ T cells occur in MM and are thought to represent MM-Ag specific T cells^35,36^. While expression of CD57 on CD8^+^, DN and CD4^+^ T cells was observed in these patient cohorts^37^, there was no evidence of CD57 expression by MAIT cells in these patients (Fig. 2Aiii and data not shown), suggesting that MAIT cells do not contribute to the CD57^+^ T cell subset in MM.

The costimulatory molecule CD27 is reduced in MM patients^35^, including in these two patient cohorts^37^ likely reflecting an increase in terminally differentiated effector memory T cells. Like DN, CD8^+^ and CD4^+^ T cells, MAIT cells had reduced CD27 expression in newly diagnosed MM patients compared to healthy controls. However, unlike CD4^+^ and CD8^+^ T cells, in which reduced CD27 expression was also evident in the R/R MM patients, CD27 expression on MAIT cells and DN T cells was observed at levels comparable with healthy controls (Fig. 2Aiv and B). This data suggests that MAIT cells are phenotypically distinct in newly diagnosed and R/R MM.

### Reduced effector function in untreated MM patients

**Figure 3:**
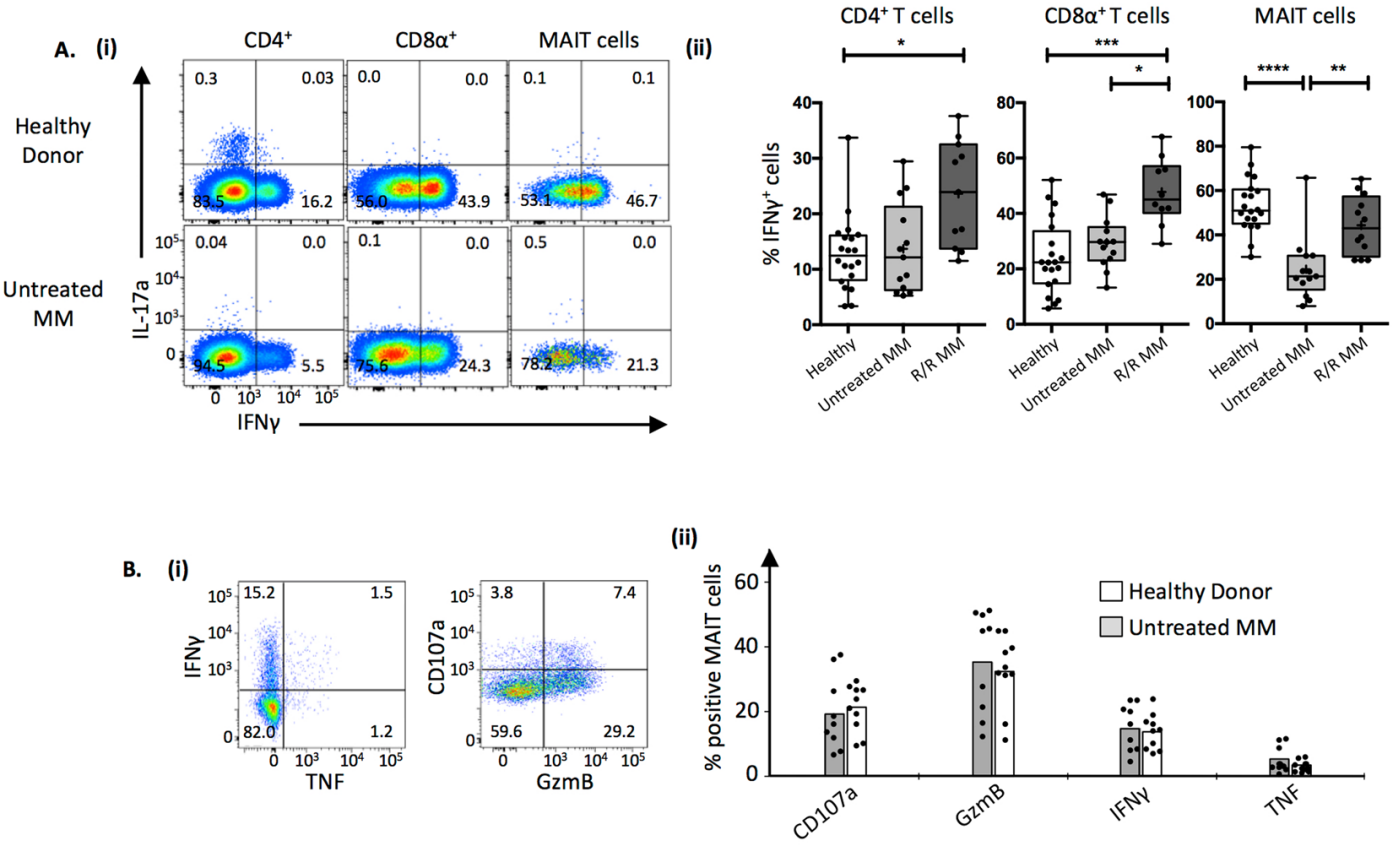
Reduced effector function by MAIT cells in multiple myeloma. (**A**) (i) Example flow cytometric pseudocolour plots showing IFNγ and IL-17A staining across T cell subsets. (ii) Box and whisker plots showing the proportion of IFNγ^+^ conventional CD4^+^ T cells, conventional CD8α^+^ T cells and MAIT cells across patient cohorts after 4 hours of PMA and ionomycin *in vitro* stimulation of PBMCs. (**B**) (i) Example flow cytometric pseudocolour plots TNF and IFNγ staining (left panel), and Granzyme B and CD107a staining (right panel) by MAIT cells after overnight co-culture of PBMCs with PFA-fixed *E. coli*. (ii) Box plots showing cumulative data derived as per B. from untreated MM patients and healthy donors. CD4^+^ and CD8^+^ T cell IFNγ expression in figure A was previously published^37^, and is reproduced here for comparison.

The effector capacity of MAIT cells was also compared between patient cohorts. PBMCs were stimulated with PMA and ionomycin, and intracellular cytokine staining (ICS) used to test for IL-17A, IFNγ and IL-22 (Fig. 3Ai and data not shown). While many MAIT cells produced IFNγ, no IL-17A or IL-22 production was detected (data not shown). CD4^+^ and CD8^+^ T cells had comparable IFNγ production in healthy donors and untreated patients whereas increased IFNγ production was seen in the R/R cohort. By comparison, MAIT cells had markedly reduced IFNγ production in the untreated cohort, which returned to normal levels in the R/R cohort (Fig. 3Aii).

As another readout of MAIT effector function, we next utilised a previously optimised bacterial stimulation assay in which PBMCs are co-cultured for 20 hours with paraformaldehyde (PFA)-fixed *Escherichia coli* (*E. coli)*, followed by flow cytometric analysis of MAIT cell effector markers^38,39^. Compared to a cohort of healthy donors, MAIT cells derived from untreated MM patients degranulated to a similar extent as determined by surface CD107a (LAMP-1) expression, and produced similar levels of granzyme B (GzmB), IFNγ and TNF (Fig. 3B). Thus, while for healthy donors a strong mitogenic stimulus such as PMA and ionomycin can greatly enhance responses compared to bacterial stimulation, this responsiveness is not enhanced in newly diagnosed MM patients, alluding to a defect in their proinflammatory capacity. This responsiveness however appears to be normal in R/R disease.

### Lenalidomide and Pomalidomide reduce MAIT cell bacterial responsiveness *in vitro*

**Figure 4:**
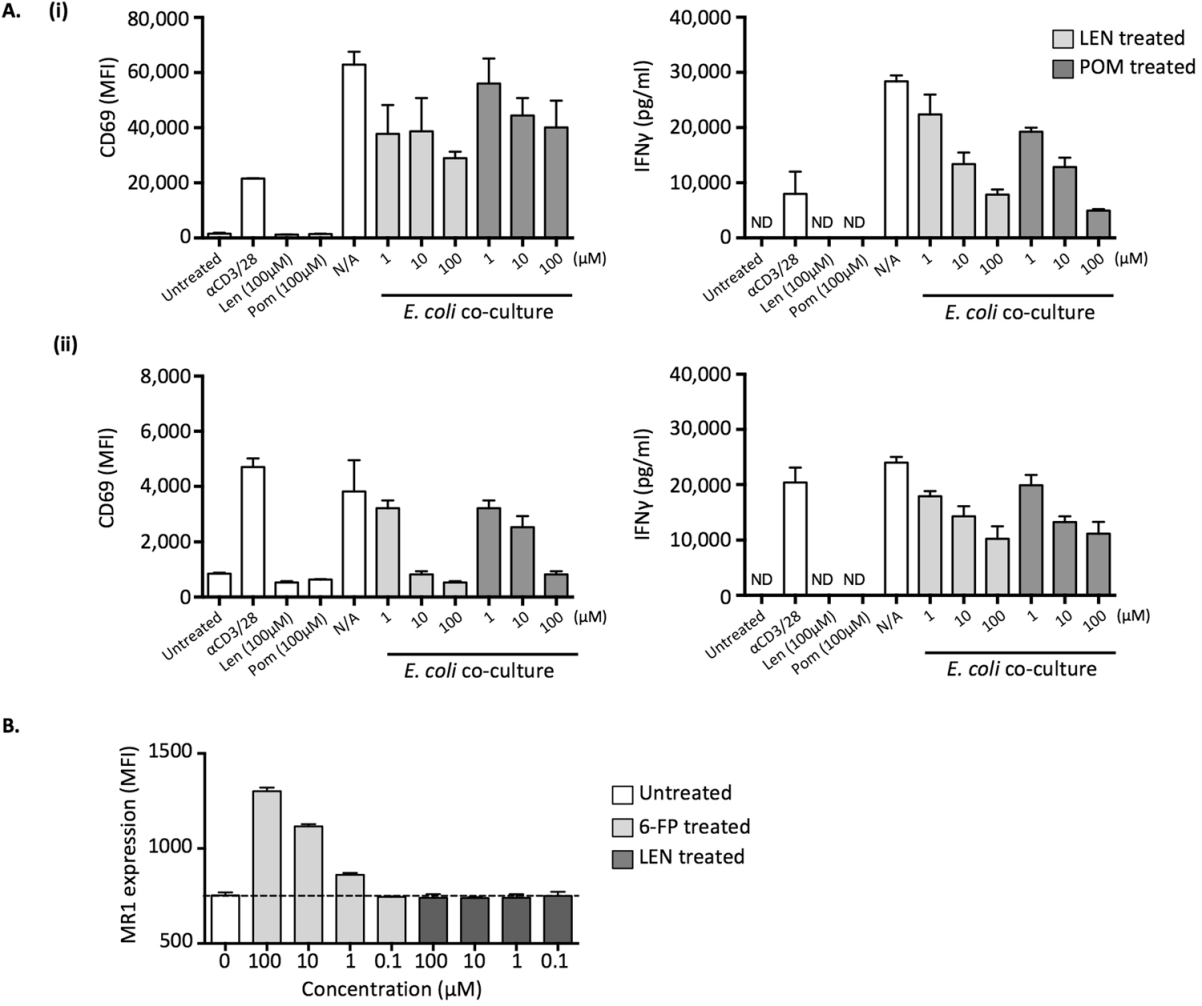
Effect of IMiDs on MAIT cell bacterial responsiveness. (**A**) Bar graphs showing (i) CD69 upregulation on MAIT cells and (ii) IFNγ present in culture supernatants in healthy donor PBMC samples cultured overnight in the presence of PFA-fixed *E. coli* for two independent experiments with different donor cells ((i) and (ii)). Error bars depict SEM of triplicate wells. (**B**) Bar graph showing MR1 expression on K562 cells treated for 4 hours in the presence of titrating quantities of 6-FP or Len. Error bars depict SEM of duplicate wells. Data is representative of 2 individual experiments.

Len, a thalidomide analogue, is an IMiD used extensively for the treatment of MM^3^. Len has previously been shown to increase IL-2 production from CD4^+^ T cells and NK cells, as well as enhancing NKT cell activity *in vitro*^40,41^, however the effect of Len on MAIT cells is unknown. To determine whether IMiDs can alter MAIT cell anti-microbial activity, we performed bacterial stimulation assays in the presence of Len and another IMID, Pom. Surprisingly, we found that MAIT cell activation was reduced by the presence of both Len and Pom in a dose-dependent manner as determined by MAIT cell CD69 upregulation, and supernatant IFNγ concentration (Fig. 4A). Given the small, cyclic structure of the IMiD compounds, one possible explanation for the reduced MAIT cell responsiveness is that the compounds bind MR1 and outcompete the bacterial ligands, preventing MR1-dependent presentation to MAIT cells. To test this, we used an established protocol previously used to screen for MR1-binding ligands^42^. K562 cells transduced with MR1 were pulsed with either 6-formyl pterin (6-FP), a folate-derivative previously shown to bind and upregulate MR1^42^, or Len. Whereas 6-FP induced dose-dependent upregulation of MR1 as expected, Len failed to modulate MR1 surface expression (Fig. 4B). Len also lacks a chemical electrophile required to form a Schiff base with Lys43 of MR1, suggesting that it is unlikely to outcompete the *E. coli*-derived covalent ligand 5-OP-RU for MR1 binding. Taken together, these data suggest that the inhibitory effects of Len and Pom on MAIT cell activation *in vitro* are independent of the MAIT TCR-MR1-Ag axis.

### Myeloma cell lines can present vitamin B metabolite Ags to MAIT cells

**Figure 5:**
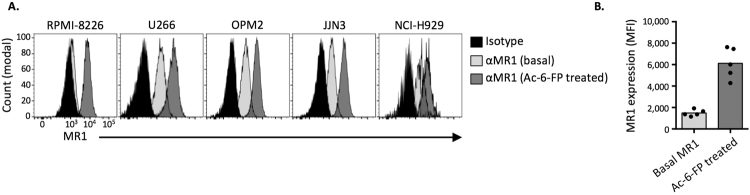
MR1 expression by myeloma cell lines. (**A**) Histogram overlays showing staining for MR1 surface expression on a panel of multiple myeloma cell lines after overnight co-culture with or without Ac-6-FP. (**B**) Bar graph representation of data plotted in A.

We next investigated whether myeloma cells may have potential as an immunotherapeutic target for MAIT cells. To determine whether myeloma cells can act as antigen presenting cells (APCs) for MAIT cells, we measured the ability of 5 different myeloma cell lines to present vitamin B-related Ags. Four out of five cell lines had detectable basal MR1 surface expression, and when pulsed with Acetyl-6-FP (Ac-6-FP), a synthetic folate derivative known to bind MR1 and induce high MR1 surface expression^42^, all 5 cell lines upregulated MR1 (Fig. 5). Consistent with reports on MR1 biology using C1R cells (a transformed B cell line)^43^, this suggests that myeloma cell lines have a basal supply of ER-resident MR1 which can rapidly egress to the surface upon binding vitamin B-derived ligands.

**Figure 6:**
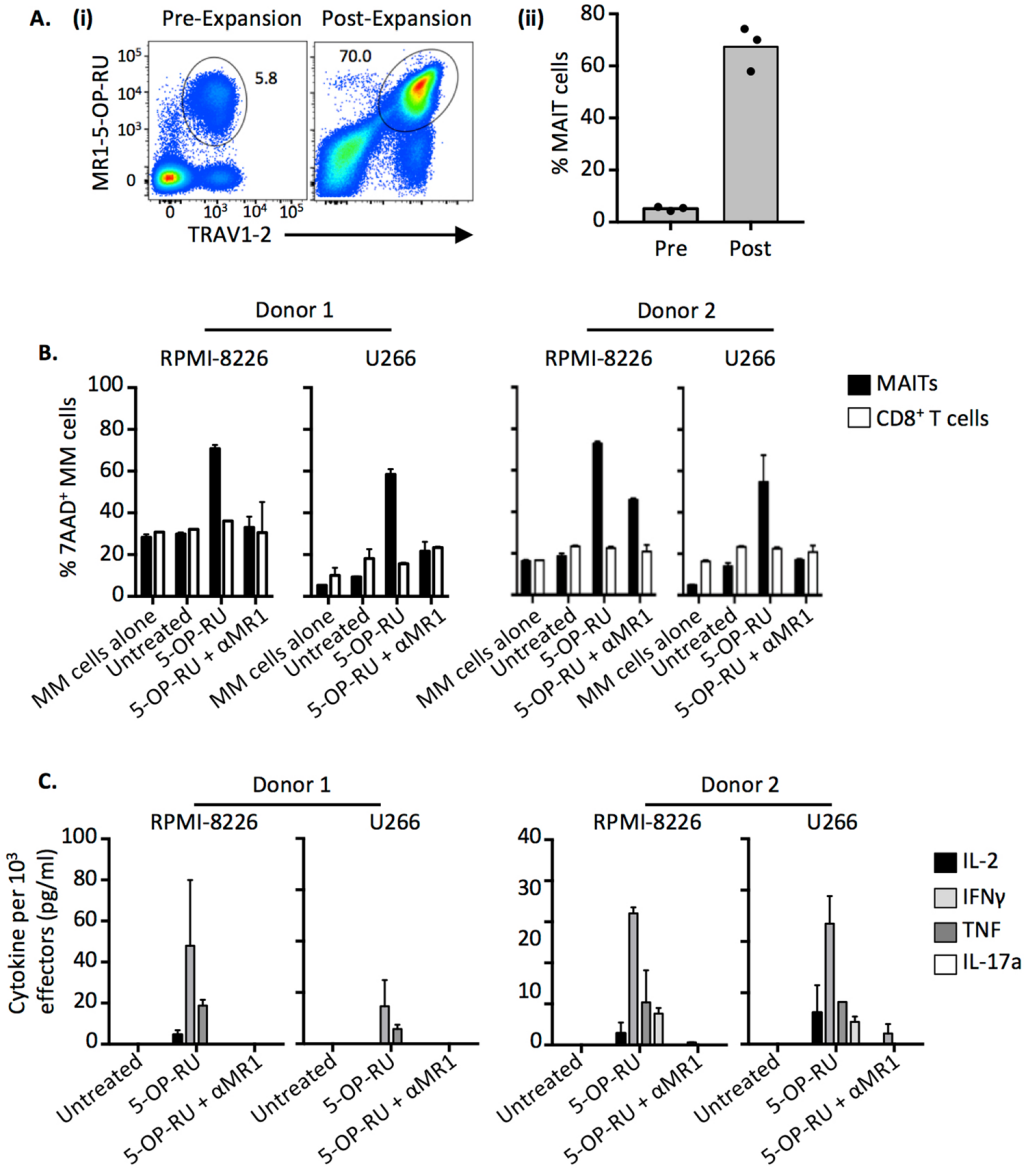
MAIT cells can detect and kill myeloma cell lines pulsed with 5-OP-RU. (**A**) (i) Example flow cytometric pseudocolour plots showing MR1-5-OP-RU tetramer staining before and after *in vitro* expansion of MAIT cells. Plots are gated on total αβ T cells. (ii) Box plots showing the proportion of MAIT cells in PBMCs directly *ex-vivo* (Pre) and after *in vitro* MAIT cell expansion (Post) from 3 individual donors as gated in A. (**B**) Box plots showing percentage cell death (7-AAD^+^ MM cells) of RPMI-8226 or U266 myeloma cell lines after overnight co-culture with sort purified *in vitro* expanded MAIT or conventional T cells at a 1:2.5 ratio from two donors. Data is representative of 3 independent experiments. Errors bars represent SEM of triplicate wells. (**C**) Box plots showing cytokine levels in culture supernatant from B. at the end of culture. Cytokine levels were divided by the number of thousand cells per well. Experiment was repeated 3 times each, on cells from 2 healthy donors in triplicate. Representative data is shown. Errors bars represent SEM of triplicate wells.

It was next important to examine whether MR1^+^ myeloma cells could be targeted by MAIT cells. In order to generate sufficient MAIT cells for *in vitro* experimentation, we were first required to expand MAIT cells *in vitro*. Due to the rarity of MAIT cells in patient samples, and the limited amount of PBMC, healthy donor PBMC samples were used for these studies. Addition of 5-OP-RU to PBMC cultures in the presence of recombinant human IL-2, IL-7, IL-12, IL-15 and IL-18 resulted in robust expansion of MAIT cells in the cultures over a 21 day period (Fig. 6A). We then purified MAIT cells and non-MAIT CD8^+^ T cells from these cultures by flow cytometric sorting, and co-cultured them *in vitro* with 2 cytogenetically distinct myeloma cell lines; RPMI-8226 and U266, in the presence or absence of 5-OP-RU antigen. Following 20 hours of co-culture, we measured myeloma cell death as determined by 7-AAD staining via flow cytometry. MAIT cells efficiently lysed both cell lines in both a 5-OP-RU and MR1-dependent manner (Fig. 6B), whereas non-MAIT CD8 T cells had no effect. These results aligned with culture supernatant cytokine levels that showed that cytokine was only produced when MAIT cells were co-cultured with the myeloma cell lines in the presence of 5-OP-RU (Fig. 6C). The MAIT cell response was largely a type I cytokine response, with IFNγ, TNF and low levels of IL-2. No IL-4, IL-5 or IL-13 was detected, but low levels of IL-17A was detected in cultures from 1 of 2 donors. Collectively, this data shows that synthetic 5-OP-RU Ag can be used for robust *in vitro* expansion of MAIT cells, and moreover that it can be efficiently presented by myeloma cell lines for recognition and induction of lytic activity by MAIT cells *in vitro*.

### Kinetics of MAIT cell killing of myeloma cells

**Figure 7:**
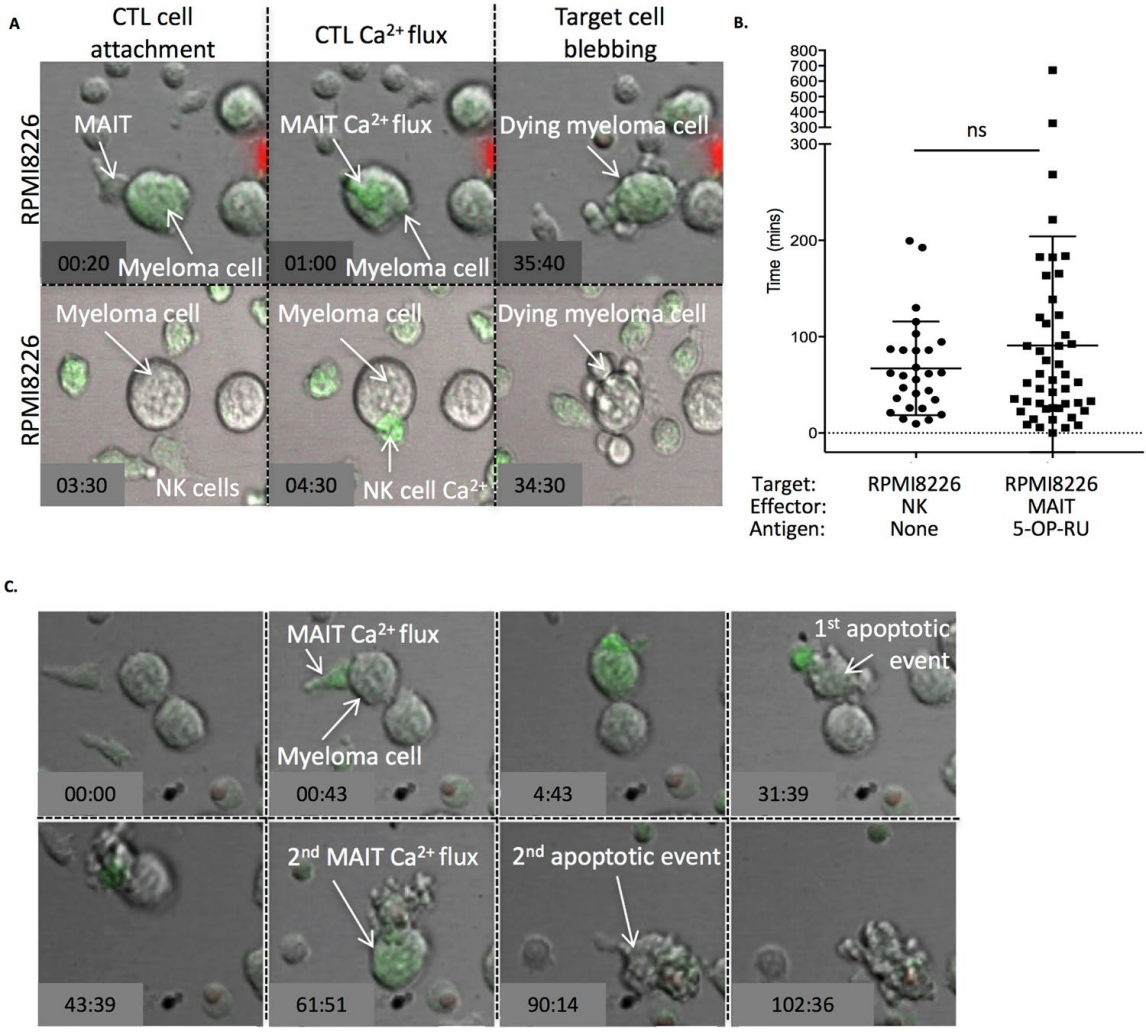
MAIT and NK cells kill myeloma cells with equivalent kinetics. Purified MAIT cells were loaded with Fluo-4-AM and co-cultured 1:1 with RPMI-8226 myeloma cells in media supplemented with 100 nM 5-OP-RU and 100uM propidium iodide. To compare MAIT cell killing kinetics of target cells, comparator co-cultures included purified NK cells co-cultured with RPMI-8226 or K562 (E:T ratio 1:1). Time lapse live video data was acquired on a Leica SP5 confocal microscope and analysed using Metamorph Imaging series 7 software. Data is presented in: (**A**) Montage of a single MAIT killing event with timestamps for MAIT attachment, MAIT Ca^2+^ flux, and myeloma cell blebbing (apoptosis), or (**B**) As collated data of individual killing events and shows the time taken (minutes) from initial contact of effector cell to target cell blebbing (apoptosis) for NK cells with K562 cells (n = 44, left), NK cells with RPMI-8226 cells (n = 28, middle) or MAIT cells with RPMI-8226 (n = 48, right). Statistical analysis (student’s t-test) was performed between test and control groups, (ns) not significant. (**C**) A video montage shows an individual MAIT cell which undergoes attachment, Ca^2+^ flux, and sequential killing of two 5-OP-RU pulsed RPMI-8226 cells.

The efficiency of MAIT cell killing of myeloma cells was next assessed at a single cell level using time lapse live video microscopy **(**Fig. 7**)**. MAIT cell killing of RPMI-8226 cells was assessed at a low magnification level in the presence or absence of 5-OP-RU Ag. MAIT cells were activated (Ca^2+^ flux), beginning from approximately 70 minutes after 5-OP-RU was added to the co-culture **(**Supplementary movie 1**)**, consistent with previous reports on the kinetics of MR1 ER-egression^43^. From this time point, MAIT cells induced apoptosis of 5-OP-RU pulsed RPMI-8226 cells, detected by myeloma cell blebbing and eventual uptake of PI **(**Supplementary movie 1**)**. In the absence of added Ag, RPMI-8226 were not recognised by MAIT cells (no green flash to indicate Ca^2+^ flux), and MAIT cell-induced RPMI-8226 apoptosis was absent (data not shown). Using higher power magnification, we assessed the kinetics of individual MAIT cell killing of 5-OP-RU pulsed RPMI-8226 cells. The time from MAIT cell attachment to Ca^2+^ flux, and the time from MAIT cell attachment to myeloma cell blebbing (apoptosis) was analysed (Fig. 7). In a representative video montage, MAIT cells attached to the 5-OP-RU pulsed RPMI-8226 cells and then underwent a Ca^2+^ flux in a short time frame (40 seconds; Fig. 7A, upper panel and Supplementary movie 2). From this time point, the MAIT cells were motile and migrated over the surface of the 5-OP-RU pulsed RPMI-8226 cells and the myeloma cells underwent blebbing (myeloma apoptosis) 35:40 minutes after the initial MAIT cell Ca^2+^ flux (Fig. 7A, upper panel and Supplementary movie 2). These killing parameters were assessed on 48 individual killing events and showed the time interval from MAIT cell attachment to myeloma cell membrane blebbing was 90.8 ± 16.5 (mean ± SEM) minutes. In addition, because the MAIT cells were motile, an accurate assessment of the precise time points of MAIT cell Ca^2+^ flux was not possible for all killing events. Thus data was plotted for all observed killing events as time from MAIT cell attachment to myeloma apoptosis.

To compare the efficiency of individual MAIT cells as killers, we co-cultured the same myeloma target cells (RPMI-8226) with purified NK cells. We observed NK cell attachment and Ca^2+^ flux, followed by RPMI-8226 blebbing/apoptosis (Fig. 7A, middle and lower panels and Supplementary movies 3–4). The time interval from NK cell attachment to RPMI-8226 killing was 67.1 ± 9.2 (mean ± SEM) minutes (Fig. 7B). There was no statistical significance in the kinetics of NK versus MAIT cell killing of RPMI-8226 cells. Finally, activated MAIT cells killed 5-OP-RU pulsed RPMI-8226 in sequential fashion (serial killing) **(**Fig. 7C and Supplementary movie 5**)**. In this representative serial killing event, the MAIT cell attached, activated and killed the 1^st^ myeloma cell (31:39 minutes), 30 minutes later the same activated MAIT cell had a 2^nd^ calcium flux attached to an adjacent myeloma cell, and induced myeloma cell apoptosis at 90:14 minutes. Thus, MAIT cell killing of targets follows similar kinetics to NK cells, and furthermore, MAIT cells are capable of serial killing.

## Discussion

MAIT cells are capable of recognising microbial metabolites as Ags, including the potent Ag 5-OP-RU, presented by MR1^20^. There is growing evidence that MAIT cells are also involved in non-infectious diseases (reviewed in^11^). Whether MAIT cells play roles in cancer pathogenesis or protection is unclear, and whether they can be harnessed as immunotherapeutic targets is also worthy of investigation. In this study, we focussed on the potential role for MAIT cells in MM and analysed the MAIT cell compartment in two cohorts of patients; those with newly diagnosed but untreated disease and those with R/R disease. MAIT cells were significantly reduced in both cohorts compared to healthy donors, with some MAIT cell subsets being more affected than others. This is reminiscent of several recent studies showing that MAIT cells are diminished in peripheral blood in a range of autoimmune, microbial, viral and metabolic diseases, as well as chronic obstructive pulmonary disease (COPD), vascular disease, fibromyalgia, asthma, hemodialysed individuals and colorectal cancer^24,25^. In some of these diseases^27–32^ MAIT cells seem to migrate from the blood to accumulate at sites of disease. In this study, we found no evidence of accumulation because MAIT cell percentages correlated between blood and BM – the active site of disease in MM.

The underlying cause of this general decline in MAIT cell numbers appears to be at least partially related to the older age of the MM patients. Little is known about the biological processes that govern MAIT cell homeostasis, however disruption of various haematopoietic processes in the myeloma BM – an anatomical location thought to be important for migratory memory T cells^44^ – may be a contributing factor. The biological significance of this decline in MAIT cells is also unclear, however given their antimicrobial activity, this may contribute to a loss of protection against infectious disease. Consistent with this notion, the likelihood of secondary infections in intensive care patients inversely correlates with the proportion of PB MAIT cells^45^. Given that infectious disease accounts for early mortality in approximately 22% of MM patients^46^, further investigation into the significance of the MAIT cell defect in the context of this disease is warranted. This is further highlighted by our novel finding that Len and Pom, immunomodulatory drugs frequently used to treat MM patients^3^, inhibited MAIT cell activation in response to bacterial stimulation.

Another recent study also analysed the frequency and number of MAIT cells in MM patients^47^. While no difference was seen in the percentage of PB MAIT cells between patients and age-matched healthy donors, the patients had a significant decrease in the absolute numbers of MAIT cells. Differences between this study and ours may be accounted for by the fact that their patient cohort was heterogeneous, both in terms of disease progression and prior treatment history. More importantly, in that study, MAIT cells were defined as CD8^+^ TRAV1–2^+^ T cells, which not only excludes DN and CD4^+^ MAIT cells from their analysis, but also includes CD161^−^ cells many of which are not MAIT cells.

Interestingly, concurrent with the decline in MAIT cell proportions, we also noted a decrease in CD27^+^ MAIT cells, specifically in the newly diagnosed patients. This aligns with similar reports in HIV patients^38^ and juvenile type I diabetes^48^ in which the CD27^−^ subset potentially represented a terminally differentiated or exhausted MAIT cell subset. Indeed, we also report a decreased ability to produce IFNγ that was specific to the newly diagnosed cohort. Whether this reduced inflammatory capacity occurs concurrently with acquisition of an immunosuppressive phenotype would be interesting to consider. This however seems unlikely given MAIT cells are universally characterised by an inflammatory transcriptional profile^21^ including expression of T-bet and RORγt, and that they were still capable of producing IFNγ albeit to a lesser degree. It nonetheless remains unclear why MAIT cells from the R/R patients exhibited normal functional potential and CD27 expression. Our previous studies of conventional T cells in these patient cohorts however, suggests that the R/R patient T cells are characterised by a heightened inflammatory capacity^37^. This observation now extends to MAIT cells. This may be reflective of a vast difference in immune status between patient cohorts at different stages of MM. Moreover, the R/R patient cohort had received a variety of previous treatments in comparison to the treatment-naïve cohort, and many of these patients had been exposed to various drugs with immunomodulatory properties, potentially influencing the phenotypic and functional capacity of the T cells in these patients. This is a highly relevant point in the context of immunotherapies. Indeed, while the effectiveness of immune-checkpoint blockade in the treatment of MM remains unclear, analysis of checkpoint molecules such as PD-1 on various T cell subsets including MAIT cells, as well as their role in immunosuppression in the MM tumour microenvironment are important to consider. For example, PDL-1/2 are expressed in the MM bone marrow including on MM cells as well as DC subsets^49^. Elevated PD-1 expression has also been reported on CD4^+^ and CD8^+^ T cells in MM patients^50^. Moreover, MAIT cells universally express PD-1 in both blood and peripheral sites^51^, and indeed this can be enhanced in disease settings^52^. MM however has far less mutational heterogeneity in comparison to checkpoint-blockade-responsive tumours such as metastatic melanoma, and thus less immunogenic potential^53^. Furthermore, initial clinical trials with anti-PD-1 in MM have been disappointing, with no objective responses in R/R patients, although minor responses have since been observed in combination with IMiDs^54^. Thus, it is unclear what the functional relevance of checkpoint molecule expression on MAIT cells in the context of MM would be, although further investigation in follow-up studies is certainly warranted.

MAIT cells have previously been shown to have cytotoxic activity against infected epithelial^55^, monocytic^22^ and embryonic kidney^56^ cell lines *in vitro*, and this activity is dependent on both MR1 as well as cytolytic mediators such as perforin and granzymes^22,55^. Our demonstration here that MM plasma cell lines can also present a synthetic Ag (5-OP-RU) to MAIT cells, and that MAIT cells can exert cytotoxic activity against these cells, provides early proof of concept that synthetic Ags may have clinical potential for tumour targeting by MAIT cells. It is important to note that the MAIT cells used in this study had been expanded *in vitro* in the presence of synthetic 5-OP-RU Ag and IL-12, IL-18, IL-7 and IL-15, which may have contributed to their cytotoxic potential^22,56,57^. Nonetheless, this supports the concept that MAIT cells can be efficiently expanded *in vitro* and then harnessed to target tumours. Further studies in this area are needed to investigate the therapeutic potential of MAIT cells, for example, using MAIT cells in adoptive T cell therapy or CAR-T cell therapy^12^.

In summary, we have shown that MM patients have low numbers of circulating MAIT cells with partially perturbed function, and hypothesise that this may be a contributing factor to the high susceptibility of MM patients to microbial disease. We also provide proof of principle that synthetic 5-OP-RU can be used to dramatically expand MAIT cells, and that these expanded MAIT cells can target and kill tumour cells. These data highlight a potential for therapeutic opportunities to manipulate MAIT cells as a form of therapy against MM and possibly other types of cancer.

## Methods

### Patient Samples

Healthy donor blood buffy coats were obtained from the Australian Red Cross Blood Service. Ethics approval was granted by both the Red Cross and the University of Melbourne Human Research and Ethics committee (1035100, 1443389). Peripheral blood mononuclear cells were isolated by standard density gradient (Ficoll-Paque Plus, GE Healthcare Life Science) and cryopreserved. Patient blood and bone marrow samples were obtained with informed concent from patients enrolled in two trials of Len plus dexamethasone treatment for either newly diagnosed MM (LITVACC trial) or relapse MM (REVLITE trial; Trial Numbers 12613000344796 and NCT00482261 respectively; details available at www.anzctr.gov.au) after approval from the Peter MacCallum Cancer Centre human ethics committee. Peripheral blood and bone marrow mononuclear cells were isolated by standard density gradient (Ficoll-Paque Plus, GE Healthcare Life Science) and cryopreserved as above. All methods used throughout were performed in accordance with the regulations and guidelines set by the regulatory bodies and ethics committees as described above.

### Previously published data

The box plots displaying CD4^+^ and CD8^+^ T cell CD27 expression in Fig. 2B, and IFNγ expression in 3A (ii) were previously published^37^. They are included here for comparison to the unpublished MAIT cell data which was acquired at the same time.

### Flow Cytometry

Unless stated otherwise, viability staining was performed in PBS prior to surface staining. Surface mAb/tetramer cocktails were then performed in 50 μl FACS buffer (PBS supplemented with 2% FBS) for 30 min at 4 °C. Cells were then washed twice and fixed in 2% paraformaldehyde (PFA) prior to acquisition on a BD LSR Fortessa. Intracellular cytokine staining was performed using a BD Cytofix/Cytoperm ICS kit as per manufacturer’s instructions. In brief, cells were surface stained as per above prior to permeabilisation with fix/perm reagent for 30 min at 4 °C. Cells were then stained with intracellular mAbs in permwash reagent for 30 min at 4 °C, washed twice with permwash and resuspended in FACS buffer ready for flow cytometric acquisition.

Surface-antigen specific mAbs included those specific for CD3ε (UCHT1, BD Biosciences), CD4 (OKT4, Biolegend), CD8α (SK1 Biolegend), CD14 (M5E2, Biolegend), CD19 (HIB19, Biolegend), CD27 (0323, Biolegend), CD33 (IV M-505, Biolegend), CD45RA (HI100, Biolegend), CD57 (NK-1, BD Biosciences), CD69 (FN50, BD Biosciences), CD161 (HP-3G10, Biolegend), IL-18Rα (H44, Biolegend), pan-TCRγδ (11F2, BD Biosciences), TRAV1-2 (3C10, Biolegend). Intracellular antigen-specific mAbs included those specific for IFNγ (4 S.B3, Biolegend), IL-17A (eBio64DEC17, eBioscience), TNF (MAb11, BD Biosciences), GzmB (GB11, BD Biosciences), CD107a (eBioHA4A4, eBioscience) and IL-22 (142928, R&D Systems). Viability dyes included the use of 7-aminoactinomycin (7AAD; Sigma), Zombie Yellow (Biolegend) and Live/Dead near infrared (Molecular Probes, ThermoFisher Scientific). Human MR1-5-OP-RU tetramers were produced in house as previously described^20^.

For analysis of clinical samples, T cells were defined as Zombie-Yellow^−^CD33^−^CD19^−^CD14^−^CD3^+^ lymphocytes (as gated by FSC-A versus SSC-A followed by doublet removal).

### Cytometric bead array (CBA)

CBA flex sets for IL-2, IL-4, IL-5, IL-13, TNF, IFNγ and IL-17A were purchased from BD Biosciences and experiments performed as per manufacturer’s instructions with an exception that 1/10 the amount of beads and detection reagents were used, and FACS buffer was used in place of official BD wash buffer (as determined by previous in-house titration experiments). In brief, sample (10 μl) was incubated with bead reagent cocktail (10 μl) for 1 hr at RT. PE-detection reagent (10 μl) was then added and incubated for a further 1 hr at RT in the dark. Beads were then washed with FACS buffer (200 μl), resuspended in FACS buffer (50 μl) and acquired immediately on BD LSR Fortessa. Data was analysed using Flowjo Software (Treestar) and Graphpad Prism.

### MAIT cell stimulation assays

For phorbol 12-myristate 13-acetate (PMA)/ionomycin-based stimulation, PBMCs were cultured for 4 hr in RF10 complete media (RPMI-1640 (Invitrogen, Life Technologies) supplemented with 10% (v/v) FBS (JRH Biosciences), 2% (v/v) Penicillin (100 U/ml), Streptomycin (100 μg/ml), Glutamax (2 mM), sodium pyruvate (1 mM), nonessential amino acids (0.1 mM), HEPES buffer (15 mM), pH7.2-7.5 (all from Invitrogen, Life Technologies) and 2-mercaptoethanol (50 μM, Sigma)) in the presence of 10ng/ml PMA, 1 μg/ml ionomycin and 1/500 Monensin (BD Biosciences). Cells were then harvested and stained as above.

For *E. coli* stimulation assays, PBMCs were co-cultured for 20 hr in human RF10 complete media in the presence of PFA-fixed DH5α *E. coli* (MOI 0.25 was determined as optimum), anti-CD107a mAb and anti-MR1 blocking mAb (clone 26.5, produced in-house). In the final 6 hr of culture, monensin (1:1500) and Brefeldin A (1:1000; both BD Biosciences) were added to the cultures. Cells were then harvested and stained as above. For Len/*E. coli* stimulation assays, no monensin was added.

### MR1 upregulation assays

For Len experiments, 15 × 10^3^ K562.MR1 cells were incubated in 100 μl RF10 complete media for 4 hr at 37 °C with titrating quantities of 6-FP (Schircks Laboratories) or Len (Celgene Inc). Cells were then harvested and stained for MR1 expression (mAb clone 26.5). Cells were immediately analysed using a BD LSRFortessa. For myeloma cell line experiments, cell lines were cultured overnight in the presence or absence of N(2)-acetyl-6-formylpterin (Ac-6-FP) and stained for MR1 the following day as above.

### MAIT cell *in vitro* expansion

Primary human MAIT cells were expanded *in vitro* for 21 days in human T cell culture media (1:1 (v/v) mix of RPMI-1640 and AIM-V (Invitrogen, Life Technologies) supplemented with 10% (v/v) FBS (JRH Biosciences), 2% (v/v) Human AB serum (Sigma) Penicillin (100 U/ml), Streptomycin (100 μg/ml), Glutamax (2 mM), sodium pyruvate (1 mM), nonessential amino acids (0.1 mM), HEPES buffer (15 mM), pH7.2-7.5 (all from Invitrogen, Life Technologies) and 2-mercaptoethanol (50 μM, Sigma) supplemented with 50 U/ml rhuIL-2 (Peprotech), 10 ng/ml rhuIL-7 (eBioscience), 50 ng/ml rhuIL-12 (Peprotech), rhuIL-15 (eBiosciences) and 50 U/ml rhuIL-18 (Peprotech). Cultures were spiked with 50 nM 5-OP-RU on days 1, 5 and 10. Cells were removed from rhuIL-12, rhuIL-18 and rhuIL-7 from day 10. On day 21 cells were checked for MAIT cell expansion by flow cytometry and aliquots cryopreserved.

### NK cell isolation

NK cells were isolated from healthy donor PBMCs using the human NK cell isolation kit (Miltenyi Biotec, Bergisch Gladbach, Germany**)** according to the manufacturer’s instructions. This negative MACS isolation protocol yields NK cells with no attached antibody for functional assays. The NK cell purity was checked (post-MACS sort) by FACS and shown to be >95% NK cells, including both CD56^hi^CD16^−^ and CD56^+^CD16^+^ subsets.

### MAIT cell killing assays

Prior to assays, expanded MAIT cells were thawed and rested overnight in RF10 complete media. The next day cells were harvested, washed once in FACS buffer and stained for 30 min on ice with mAbs directed against CD3, CD161, TRAV1-2 as well as 7AAD. Cells were then washed once with FACS buffer and sorted using an ARIAIII cell sorted (BD) for MAIT cells (CD3^+^, CD161^HI^, TRAV1-2^+^) and conventional T cells (CD3^+^, CD161^−^, TRAV1-2^−^). Cells were washed once in RF10 media and cultured for 20 hours at a 2.5:1 ratio of effectors:targets in 50 μl RF10 media in the presence of 100 nM 5-OP-RU and/or 40 μg/ml MR1 blocking mAb (clone 26.5). After culture, cells were harvested, washed once with FACS buffer and resuspended in 60 μl FACS buffer with 7AAD. Cells were immediately acquired with a BD LSRFortessa flow cytometer.

### Cell lines

Human myeloma cell lines RPMI8226, U266 and NCI-H929 cell lines were obtained from American Type Cell Collection (ATCC); OPM-2, and JJN-03 cell lines were from DSMZ (Braunschweig, Germany).

For production of K562.MR1 cells, the full length human MR1A gene was cloned into a pMIG expression vector. The pMIG vector was then used to stably transfect wild type K562 cells by retroviral transduction using HEK293T cells as packaging cells as previously described^58^. Cells were then sort purified for high MR1 expression using an ARIA-III cell sorted (BD) to produce stable cell lines.

### Live Cell Microscopy

*In vitro* expanded human MAIT cells were sort purified as above and NK cells were purified from healthy donors as above. These effector cells were then assessed for efficiency of myeloma cell killing by time lapse live video microscopy using a modification of a previously published protocol^59^. RPMI-8226 or K562 cells were seeded at 3 × 10^4^ cells in each well of an 8-well chamber slide (ibidi, Munich, Germany) and incubating overnight at 37 °C/10% CO_2_.

Purified MAIT cells were pre-loaded with Fluo-4-AM (20 min with 1 μM Fluo-4 and 0.02% [w/v] Pluronic F-127 carrier at 37 °C/10% CO_2_). MAIT cells were co-cultured 1:1 with RPMI-8226 myeloma cells in media supplemented with 100 nM 5-OP-RU and 100 μM propidium iodide. Control co-cultures included purified NK cells co-cultured with RPMI-8226 or K562 (E:T ratio 1:1). Chamber slides were mounted on a heated stage within a temperature-controlled chamber maintained at 37 °C, and constant CO_2_ concentrations (5 or 7% “The Brick”; ibidi, Munich, Germany). Optical sections were acquired through sequential scans of Fluo-4 (excitation 488 nm), propidium iodide (excitation 561 nm), or Brightfield/DIC on a Leica SP5 confocal microscope (Leica Microsystems, Deerfield, IL) using a 40X(NA 0.85) air objective and Leica LAS AF software. For the 488 and 561 channels, the pinhole was set to 4.2 AU, giving a section thickness of 5 µM and XY pixel size of 378.8 nM. Images were acquired every 20 seconds, video data was analysed using Metamorph Imaging series 7 software (Universal Imaging).

### Statistical analysis

All statistical analysis was performed using Graphpad Prism. Statistical tests comparing 2 non-paired patient cohorts used Mann-Whitney tests, tests comparing 3 non-paired patient cohorts used Kruskal-Wallis tests and tests comparing patient-matched peripheral blood and bone marrow used Wilcoxon matched-pairs signed rank tests. A student t test was used to compare two large sample size groups in Figure 7 (*0.05 > p > 0.05; **0.05 > p > 0.005, ***0.005 > p > 0.0005, ****0.0005 > p). For box and whisker plots, individual data points are depicted, boxes represent upper quartile, median and lower quartile, whiskers represent minimum and maximum values and plus sign represents mean.

The datasets generated and/or analysed during the current study are available from the corresponding author on reasonable request.

## Electronic supplementary material

Supplementary Movie 1

Supplementary Movie 2

Supplementary Movie 3

Supplementary Movie 4
